# Prevalence of Iatrogenic Vitamin D Toxicity Among the Saudi Population of Vitamin D Users Due to Overcorrection

**DOI:** 10.7759/cureus.37521

**Published:** 2023-04-13

**Authors:** Faisal Aljehani, Mohammed B Qashqari, Mohammed K Alghamdi, Abdalaziz I Saadi, Mohamed Y Alreasini, Enad Alsolami, Mohammed Alfawaz

**Affiliations:** 1 Internal Medicine, College of Medicine, University of Jeddah, Jeddah, SAU

**Keywords:** overdose, vitamin d, misuse, saudi arabia, hypercalcemia

## Abstract

Background

Despite abundant sunlight exposure, vitamin D deficiency remains a major challenge in Saudi Arabia. Meanwhile, the widespread use of vitamin D supplements has prompted concerns about toxicity, which although rare, can have severe health consequences.

Objective

The objective of this cross-sectional study was to analyze the prevalence and associated factors of iatrogenic vitamin D toxicity among the Saudi population of vitamin D users due to overcorrection.

Methods

An online questionnaire was used to collect data from 1,677 participants across all regions of Saudi Arabia. The questionnaire included responses on the prescription, duration of vitamin D intake, dosage, frequency, history of vitamin D toxicity, symptom onset, and duration.

Results

One thousand six hundred and seventy-seven responses were included across all regions of Saudi Arabia. A majority of participants were female (66.7%) and around half were aged 18-25 years. A history of vitamin D use was reported by 63.8% of participants, and 48% were still using vitamin D supplements. Most participants (79.3%) consulted a physician, and 84.8% had taken a vitamin D test before using the supplement. Commonly reported motives for taking vitamin D included vitamin D deficiency (72.1%), lack of sun exposure (26.1%), and hair loss (20.6%). Symptoms of overdose were reported by 6.6% of participants, with 3.3% having an overdose and 2.1% experiencing both overdose and symptoms.

Conclusion

This study showed that although a large portion of the Saudi population is taking vitamin D supplements, the prevalence of vitamin D toxicity is relatively low. However, this prevalence should not be ignored, and further research is needed to understand the factors contributing to vitamin D toxicity in order to minimize its occurrence.

## Introduction

Vitamin D plays an important role in regulating calcium and phosphate metabolism [1]. However, emerging research into its potential biological functions has uncovered that it has effects beyond the skeletal system. The active form of vitamin D also exhibits immunological properties [2]. These findings have resulted in widespread vitamin D supplementation. Despite ample sunlight exposure in Saudi Arabia, vitamin D deficiency is still prevalent. Numerous studies have reported a high prevalence of vitamin D deficiencies across Europe [3-4]. In Saudi Arabia, research shows that 63.2% of the population is vitamin D deficient, with a higher proportion of women (62%) than men (38%) [5]. Furthermore, a study conducted in Riyadh, Saudi Arabia, reported that only 20% of Saudi women had sufficient vitamin D [6]. Another study reported a high prevalence of vitamin D deficiency among young adults and those with central obesity, with 72% of males and 64% of females having insufficient vitamin D levels [7].

While vitamin D supplementation is vital for addressing the deficiency, the challenge of vitamin D toxicity has emerged with the increase in prescriptions. A study conducted in a tertiary care center in India from January 2011 to January 2013 revealed that the prescription of high doses of vitamin D caused vitamin D toxicity [8]. A survey of Saudi dermatologists indicated that 60% of dermatologists in Saudi Arabia prescribe vitamins and minerals for hair loss treatment [9]. To avoid vitamin D toxicity, it is recommended to use between 800 and 1000 IU/day of vitamin D to alleviate vitamin D deficiency [10]. Vitamin D toxicity can be caused by prescription errors, high-dose preparations, or incorrect dosing.

A noteworthy finding is that almost 75% of published reports on vitamin D toxicity have surfaced after 2010 [11]. The clinical manifestations of vitamin D toxicity commonly include nausea, vomiting, dehydration, and loss of appetite, while hypercalciuria and hypercalcemia [12].

The growing awareness of the health benefits of vitamin D has led to an increase in the use of vitamin D supplements among the Saudi population, either through prescriptions or over-the-counter. However, there is a lack of research on the incidence of vitamin D toxicity in this population. This study aimed to fill this gap by measuring the prevalence of vitamin D toxicity resulting from overcorrection in Saudi patients.

## Materials and methods

Study design

This cross-sectional study aims to identify the prevalence and associated factors of vitamin D overdose and toxicity. The study used a questionnaire tested and validated by a team of experts. 

Study area

The study was conducted in the region of the Kingdom of Saudi Arabia, which has over 35 million people. Saudi Arabia has 13 administrative-territorial entities, which were included in our study. 

Study population

Saudi individuals aged ≥18 years old who have access to the internet and can participate are eligible for inclusion. Individuals using supplements other than vitamin D, non-Saudis, and uncompleted forms are not eligible for this study. While the calculated sample size was 384 participants, the authors reached 1,677 responses for analysis. Convenient sampling was used.

Data collection

A structured questionnaire (Appendix 1) form was designed using an online tool (Google Forms) to reach the participants. The online form contained four sub-divisions sections that were demonstrated according to each participant’s responses. The first section contained information about the study’s objectives for obtaining consent. The second section inquired about sociodemographic characteristics. The third section inquired about the usage of vitamin D supplements, including the presence of a prescription, the duration of use, performing laboratory tests, frequency, dosage, and reason for taking vitamin D supplements. The last section included questions about overdose history, symptom onset, duration, and laboratory test findings.

Statistical analysis 

The statistical package for the social sciences (IBM Corp. Released 2020. IBM SPSS Statistics for Windows, Version 27.0. Armonk, NY: IBM Corp) was used to analyze the data. As the questions on the forms were optional, missed values among different variables were present. This was not managed for questions exceeding 5% missing values and was computed as missing during the analysis. Proportions and contingency tables were used to summarize the categorical variables. The outcome variable was adjusted to be binary (having either overdose symptoms or not), which was tested for significance with independent variables. Chi-square and Fisher’s exact tests were used to find significant associations with the outcome variable. The significance level was set at 0.05.

Ethical considerations

The current study was approved by the research ethics committee of Fakeeh College of Medical Sciences, approval number 271/IRB/2022. This study used voluntary and anonymous participation without collecting personal identifiers to protect the participants’ confidentiality. Furthermore, the collected data is used for research purposes only. All responses were collected only after explaining the study objectives and after obtaining the consent of each participant, which was a required section, before answering the questions.

## Results

A total of 1,677 responses were analyzed. About half (47.3%) were in the 18-25 age group, and two-thirds (66.7%) were females. All the regions of Saudi Arabia were included, with Riyadh and Makkah representing the most frequent regions, with 24.4% and 24.3%, respectively. Bachelor’s and high school were also the most frequently indicated educational levels. The sociodemographic distribution of the participants is shown in Table 1.

**Table 1:** Sociodemographic characteristics of the participants

		N	%
Age	18-25	793	47.3%
	26-35	415	24.7%
	36-45	274	16.3%
	46-55	147	8.8%
	56-65	38	2.3%
	66-75	6	0.4%
	>75	4	0.2%
Gender	Male	559	33.3%
	Female	1118	66.7%
Region	Riyadh	410	24.4%
	Makkah	407	24.3%
	Madinah	86	5.1%
	Eastern	187	11.2%
	Tabuk	90	5.4%
	Qassim	67	4.0%
	Aseer	49	2.9%
	Hail	35	2.1%
	Baha	46	2.7%
	Jazan	91	5.4%
	Najran	58	3.5%
	Jouf	96	5.7%
	Northrn	55	3.3%
Educational level	None	10	0.6%
	Elementary	0	0.0%
	Secondary	75	4.5%
	High school	560	33.4%
	Diploma	168	10.0%
	Bachelor	775	46.2%
	Master	72	4.3%
	PhD	17	1.0%

**Figure 1:**
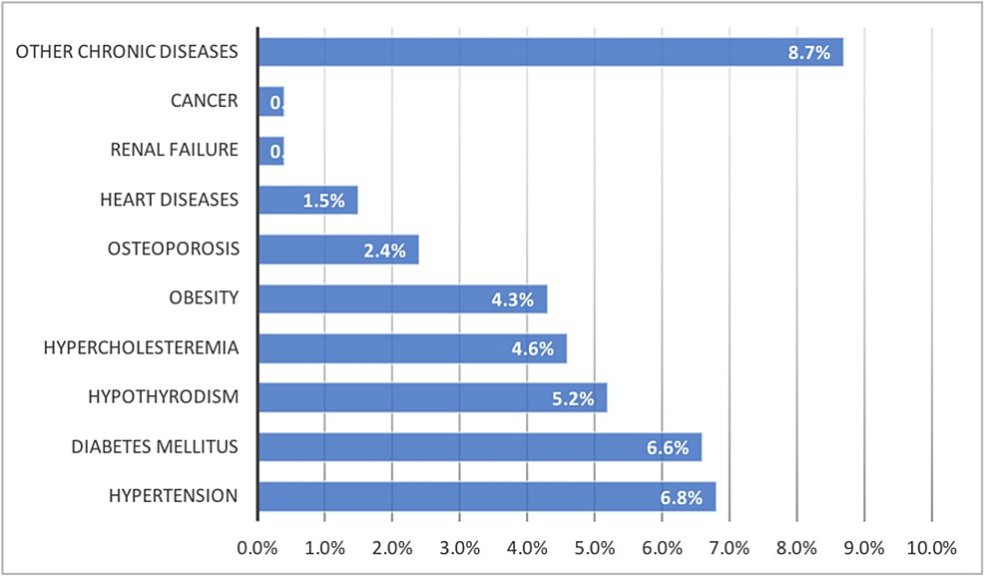
Chronic diseases among the participants

Of the total participants in the survey, 6.8% had hypertension, 6.6% had diabetes, 5.2% had hypothyroidism, 4.6% had hypercholesteremia, 4.3% had obesity, 2.4% had osteoporosis, 1.5% had cardiovascular disease, 0.4% had cancer, 0.4% had renal failure, and 8.7% had other diseases. The presence of chronic diseases is demonstrated in Figure.1. 

A history of using vitamin D supplements was reported by 63.8% of the study participants, of whom 48% are still currently taking vitamin D supplements. The majority of participants (79.3%) have consulted with a physician regarding their vitamin D supplement use, and 84.8% have had a vitamin D test before starting the supplements. Among those who have used vitamin D supplements, 28.7% used them for less than a month, 41.7% for one to three months, 13.1% for more than three to six months, 7.7% for more than six months to one year, and 8.9% for more than one year. The most common dosing interval was weekly (62.8%), followed by daily and monthly (20.6% and 12.8%, respectively). Among daily users, 125 IU was the most common dose (30.9%), while among weekly and monthly users, the most common dose was 50,000 IU, with 33.2% and 31.4% respectively. The frequency and doses are presented in Table 2.

**Table 2:** The frequency and doses of vitamin D supplements among the participants

		Frequency							
		Daily		Weekly		Monthly		Other	
		N	%	N	%	N	%	N	%
Dose	125 IU	68	30.9%	0	0.0%	0	0.0%	11	27.5%
	150 IU	22	10.0%	0	0.0%	0	0.0%	2	5.0%
	320 IU	8	3.6%	0	0.0%	0	0.0%	0	0.0%
	400 IU	11	5.0%	0	0.0%	0	0.0%	2	5.0%
	600 IU	8	3.6%	85	12.6%	0	0.0%	0	0.0%
	800 IU	5	2.3%	14	2.1%	0	0.0%	1	2.5%
	1,000 IU	46	20.9%	57	8.5%	19	13.9%	4	10.0%
	2,000 IU	3	1.4%	8	1.2%	5	3.6%	0	0.0%
	2,800 IU	7	3.2%	8	1.2%	0	0.0%	0	0.0%
	5,000 IU	29	13.2%	132	19.6%	29	21.2%	8	20.0%
	10,000	10	4.5%	19	2.8%	6	4.4%	0	0.0%
	> 10,000 IU	3	1.4%	0	0.0%	0	0.0%	0	0.0%
	<600 IU	0	0.0%	115	17.1%	0	0.0%	0	0.0%
	50,000 IU	0	0.0%	223	33.2%	43	31.4%	10	25.0%
	>50,000 IU	0	0.0%	11	1.6%	0	0.0%	0	0.0%
	<1,000 IU	0	0.0%	0	0.0%	34	24.8%	0	0.0%
	100 IU	0	0.0%	0	0.0%	1	0.7%	1	2.5%
	>100,000 IU	0	0.0%	0	0.0%	0	0.0%	1	2.5%

Participants were asked about their reasons for taking the supplements. The most common reasons reported were having a vitamin D deficiency confirmed by a blood test (72.1%), not getting enough sun exposure (26.1%), and hair loss (20.6%). The reasons for taking supplements are shown in Table 3.

**Table 3:** Reasons for taking vitamin D supplements among the participants

	Yes		No	
	N	%	N	%
Personal choice	101	9.4%	969	90.6%
Advise from a relative‎\friend	83	7.8%	987	92.2%
The test showed vitamin D deficiency	772	72.1%	298	27.9%
For muscle growth	46	4.3%	1024	95.7%
For hair loss	220	20.6%	850	79.4%
To strengthen my immunity	164	15.3%	906	84.7%
To strengthen my bones	150	14.0%	920	86.0%
I don't get enogh sun exposure	279	26.1%	791	73.9%
I am pregnant	29	2.7%	1041	97.3%
I am using vitamin D for other reasons	17	1.6%	1053	98.4%

**Figure 2:**
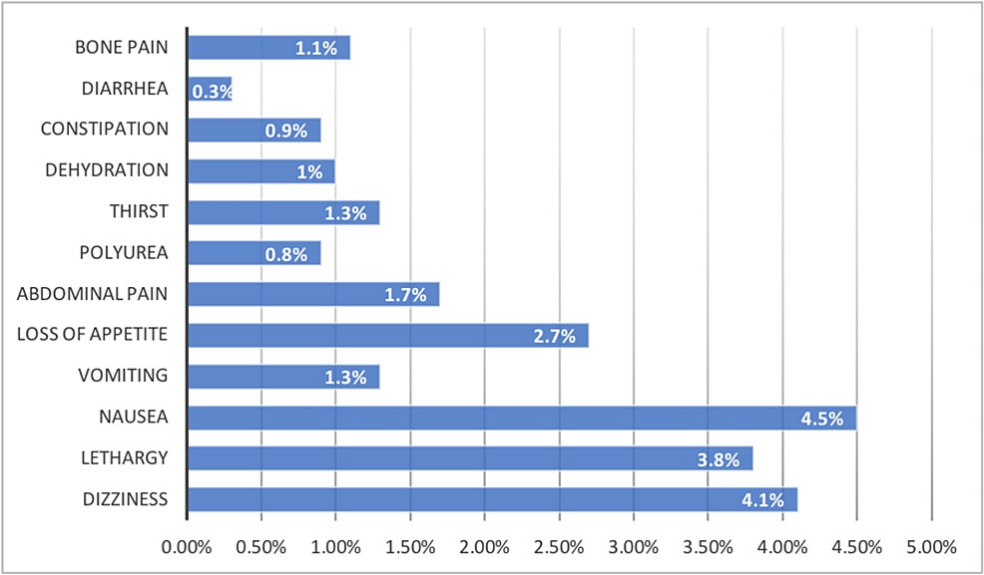
The distribution of symptoms among those who developed vitamin D toxicity

The participants were asked whether they had experienced an overdose or symptoms as a result of taking vitamin D supplements. The responses revealed that 6.6% had reported symptoms, 3.3% had taken an overdose, 2.1% had experienced both an overdose and symptoms and 88% had either an overdose or symptoms. The symptoms reported by participants included nausea (4.5%), dizziness (4.1%), lethargy (3.8%), loss of appetite (2.7%), abdominal pain (1.7%), thirst (1.3%), vomiting (1.3%), bone pain (1.1%), dehydration (1%), constipation (0.9%), polyurea (0.8%), and diarrhea (0.3%). The proportions of symptoms reported by the participants are demonstrated in Figure 2. Of those who had either an overdose or symptoms, 44.1% had undergone laboratory tests, which showed toxicity in 41.5%, deficiency in 39%, and normal levels in 19.5%.

Among those who have experienced a vitamin D overdose, 45.6% reported experiencing it once, 26.3% twice, 12.3% three times, 8.8% four times, and more than four times by 7%. More than half (57.9%) of them have visited a doctor after an episode of vitamin D overdose. The symptoms appeared within six hours in 52.7% of those who had them, within six to twelve hours in 19.4%, within more than twelve hours to one day in 8.6%, and within more than one day in 19.4%. The duration of symptoms was 30 minutes among 14%, more than 30 to 60 minutes among 31.2%, more than one hour to two hours among 18.3%, and more than two hours among 36.6%.

Toxicity and symptoms were categorized as a binary variable representing the outcome of having either an overdose or symptoms. This variable was further tested for significance with age, gender, and educational level, but no significant associations were found. The results are shown in Table 4.

**Table 4:** Vitamin D overdose distribution by sociodemographic characteristics

		Overdose or symptoms				
		Yes		No		P-value
		N	%	N	%	
Age	18-25	44	9.9%	401	90.1%	0.601
	26-35	21	7.5%	258	92.5%	
	36-45	17	9.9%	155	90.1%	
	46-55	9	8.7%	95	91.3%	
	56-65	1	3.4%	28	96.6%	
	66-75	1	25.0%	3	75.0%	
	>75	0	0.0%	2	100.0%	
Gender	Male	28	9.1%	281	90.9%	0.520
	Female	65	9.0%	661	91.0%	
Educational level	None	1	20.0%	4	80.0%	0.372
	Elementary	0	0.0%	0	0.0%	
	Secondary	5	10.4%	43	89.6%	
	High school	35	11.4%	271	88.6%	
	Diploma	8	7.6%	97	92.4%	
	Bachelor	37	7.4%	464	92.6%	
	Master	6	10.7%	50	89.3%	
	PhD	1	7.1%	13	92.9%	

Table 5 summarizes the results of further analyses that investigated the associations between the overdose/symptoms and the comorbidities and reasons for taking vitamin D supplements. The analysis revealed that hypercholesteremia, other chronic diseases, and still taking vitamin D were significantly associated with having an overdose or symptoms. Additionally, several motives for taking vitamin D supplements, including assistance from a relative or friend, tests showing vitamin D deficiency, hair loss, strengthened immunity, and strengthening bones, were found to be statistically significant. 

**Table 5:** Variables that were significantly associated with vitamin D overdose

		Overdose or symptoms				
		Yes		No		P-value
		N	%	N	%	
Hypercholesteremia	Yes	9	19.1%	38	80.9%	0.031
	No	84	8.5%	904	91.5%	
Other chronic diseases	Yes	14	15.9%	74	84.1%	0.029
	No	79	8.3%	868	91.7%	
Are still taking vitamin D?	Yes	56	11.2%	442	88.8%	0.017
	No	37	6.9%	500	93.1%	
Advise from a relative‎\friend	Yes	17	22.4%	59	77.6%	<0.001
	No	76	7.9%	883	92.1%	
The test showed vitamin D deficiency	Yes	55	7.2%	708	92.8%	0.001
	No	38	14.0%	234	86.0%	
For hair loss	Yes	28	13.2%	184	86.8%	0.021
	No	65	7.9%	758	92.1%	
To strengthen my immunity	Yes	22	14.2%	133	85.8%	0.021
	No	71	8.1%	809	91.9%	
To strengthen my bones	Yes	26	17.9%	119	82.1%	<0.001
	No	67	7.5%	823	92.5%	

## Discussion

Vitamin D plays a crucial role in various cellular processes, such as immune system modulation, calcium homeostasis, and cell differentiation [13]. Despite abundant sunlight exposure in MENA (Middle East and North Africa) regions, hypovitaminosis D remains a significant concern for the population. A review study on vitamin D deficiency reported a prevalence of 54%-90% among pregnant women and 44%-96% among adults. To correct deficiencies, a dose of 1000-2000 IU/d is recommended to achieve optimal vitamin D levels of 20 ng/ml in the MENA regions [14]. A meta-analysis conducted between 2008 and 2015 reported a 60% vitamin D deficiency among the Saudi Arabian population [5]. While vitamin D supplementation is advised to treat vitamin D deficiency, high levels can also have negative consequences, including toxicity. This study aims to assess vitamin D toxicity among the Saudi Arabian population due to overcorrection

Most participants (63.8%) had a history of using vitamin D supplements, with 48% still taking them. The daily vitamin D intake was 13.1% among the participants, which was lower than the study conducted in Riyadh which reported 29.5% of participants taking vitamin D daily, with 60.2% of women having hypovitaminosis D [6]. Even the minimum dose (400 IU/day) was higher than the current study, where only 55% were taking 400 IU/day or more. Our findings were consistent with a study from the Qassim region of Saudi Arabia, where only 18.8% were taking vitamin D [15]. A cross-sectional study in the United States reported higher vitamin D supplementation rates (36.6%), but most participants lived in low-UV exposure areas, which explains their higher vitamin D intake. The study also revealed the relationship between sun exposure and vitamin intake, where 26.1% of participants reported a lack of sun exposure as their reason for taking vitamin D. Recent findings have shown that vitamin D is essential for skeletal and immune health. In this study, 15.3% of participants reported taking vitamin D to boost their immunity [16].

The majority of participants (79.3%) in our study had consulted a doctor before starting on vitamin D. These findings are in contrast to another study that reported only 6.3% taking vitamin D under medical supervision [17]. The common reasons for taking the supplement included confirmed deficiency, lack of sun exposure, and hair loss. In India, a study reported backache, radiculopathy, and osteoarthritis as the reasons for supplement intake [18]. Vitamin D plays a crucial role in the growth and differentiation of hair follicles. Multiple studies have reported a correlation between serum vitamin D levels and alopecias, such as alopecia areata, androgenetic alopecia, and scarring alopecia [19]. About 20.6% of participants reported hair loss as the reason for taking vitamin D. Another study in 2016 also reported a relationship between female pattern hair loss and low levels of vitamin D [20].

The current study revealed that 6.6% of participants had symptoms, 3.3% had an overdose, and 2.1% had both symptoms and an overdose. Similar findings were reported in 2016, with 8.6% of participants having concentrations of >500 nmol/L, which is considered toxic [17]. A 16-year retrospective study found that only 1.05% of people had serum vitamin D levels higher than 80 ng/ml, and only 0.12% had levels higher than 120 ng/ml. Only four participants showed signs of toxicity [21]. The symptoms of vitamin D toxicity can range from thirst and discomfort to severe symptoms, including seizures and coma [11]. Nausea (4.5%), dizziness (4.1%), lethargy (3.8%), abdominal pain (1.7%), and vomiting (1.3%) were the main clinical features reported among participants in the current study. These findings are supported by a study in 2017, which reported clinical manifestations such as nausea, vomiting, altered sensorium, and weight loss in cases of vitamin D toxicity in Kashmir, India [22].

Self-medication with vitamin D, without the supervision of a professional healthcare specialist, poses a serious risk of toxicity. In the present study, 22.4% of respondents who took vitamin D on the advice of friends or relatives reported toxicity. Multiple case reports support these findings, including a case report of a female patient who presented with vomiting, severe abdominal pain, and worsening palpitations. After a thorough examination, hypervitaminosis D was diagnosed and corrected [23]. Similar findings were reported from the Columbia University Medical Center, where nine patients presented with hypercalcemia symptoms. Upon evaluation, it was revealed that they took over-the-counter vitamin supplements that had more than the labeled dose of vitamin D [24]. These findings suggest a stronger link between self-medication and vitamin D toxicity.

One of the major strengths of this study is its novelty, as it is one of the few studies conducted on the subject of vitamin D toxicity and overdose in Saudi Arabia and the Middle East. The large sample size of 1677 participants, who were recruited from all over Saudi Arabia, also adds to the strength of this study. Moreover, the use of an online survey made it easier to reach a diverse population, and the convenience sampling method ensured a broad representation of the population.

One of the limitations of this study is the use of convenience sampling, which may limit the generalizability of the findings to the entire population of Saudi Arabia. Additionally, the use of self-reported data may have introduced some biases, such as recall bias and social desirability bias. Moreover, since the study relied on an online survey, it may have excluded individuals who do not have access to the internet or are less familiar with the technology. Finally, the study did not include a clinical assessment of vitamin D toxicity or overdose and instead relied on self-reported symptoms and self-reported laboratory tests, which may not always be accurate or reliable. Future studies should consider incorporating clinical examinations and laboratory tests to more accurately determine the prevalence of vitamin D toxicity and its associated factors, thereby providing a more definitive understanding of iatrogenic toxicity.

## Conclusions

Given the broad therapeutic applications of Vitamin D, overdose has emerged as a significant issue. Historically, manufacturing errors, inappropriate administration, and incorrect prescriptions have been the primary contributors to vitamin toxicity. This study sheds light on the high prevalence of vitamin D intake among the Saudi population, with 13.1% taking it daily. Moreover, most participants consulted with their doctors before taking vitamin D, highlighting the importance of professional guidance to minimize vitamin D toxicity. The study found that few participants experienced vitamin D overdose and its symptoms. Overall, this study has provided valuable insights into the issue of vitamin D toxicity, which can be used to develop strategies for reducing the incidence of vitamin D overdoses.

## Figures and Tables

**Figure 3:**
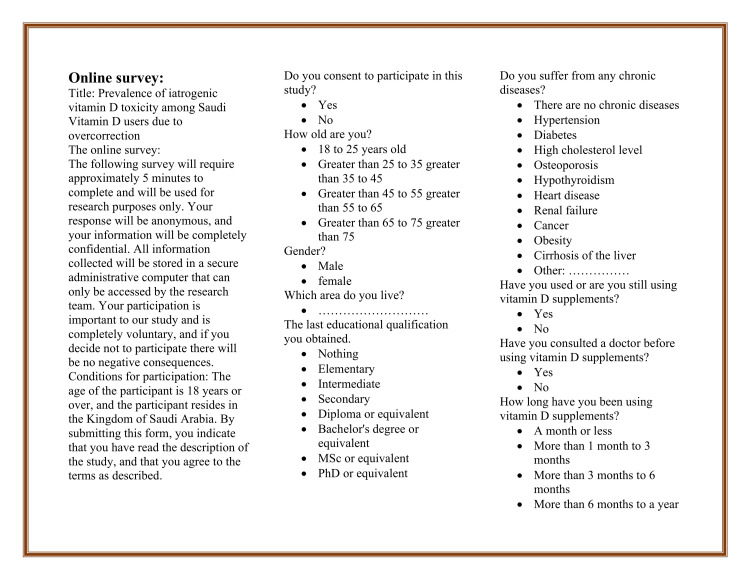
Page 1 of the online survey

**Figure 4:**
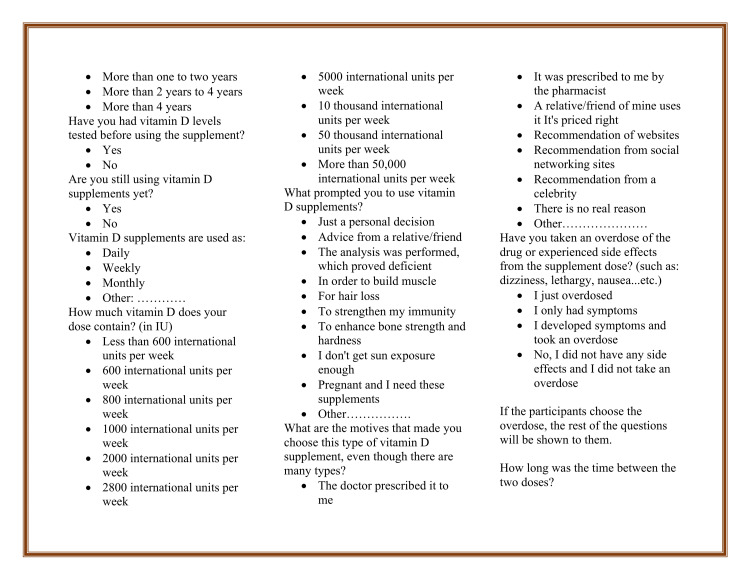
Page 2 of the online survey

**Figure 5:**
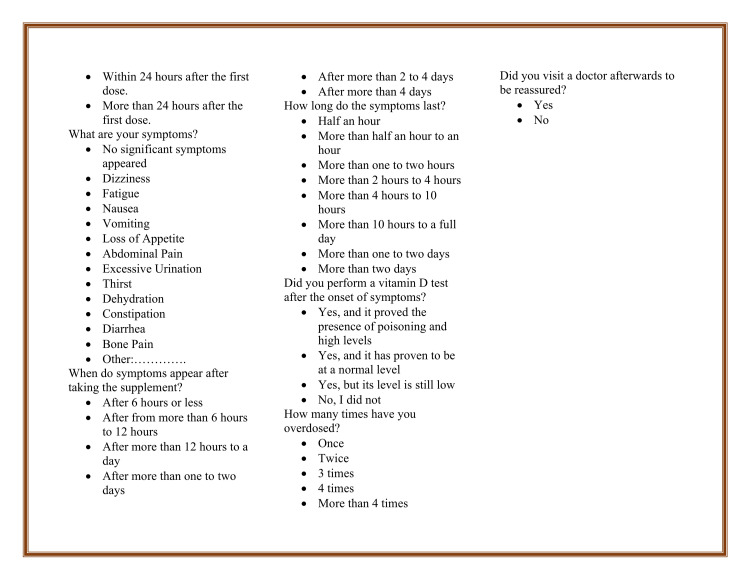
Page 3 of the online survey

## Appendices

Appendix 1

Online survey questions are shown in Figures 3, 4 and 5.
